# Editorial: Molecular biology of biomarkers in diagnosis and treatment of glioblastoma multiforme

**DOI:** 10.3389/fonc.2023.1132628

**Published:** 2023-01-30

**Authors:** Jianyi Zhao, Yingying Lin

**Affiliations:** ^1^ Brain Injury Center, Shanghai Institute of Head Trauma, Ren Ji Hospital, Shanghai Jiao Tong University School of Medicine, Shanghai, China; ^2^ Shanghai Cancer Institute, Ren Ji Hospital, Shanghai Jiao Tong University School of Medicine, Shanghai, China

**Keywords:** glioblastoma, prognosis, biomarker, pathophysiological mechanisms, nomograms, immune

Glioblastoma (GBM) is the most common malignant brain tumor in the central nervous system with high mortality and morbidity. The annual incidence of GBM is about 3-6/100,000 people, with a median post-diagnostic survival time of 14.6 months and a 5-year survival rate of 5.6% (1–3). Initial surgical resection, adjuvant chemoradiotherapy with temozolomide constitute the standard-of-care therapy for GBM, while surgical resection alone provides a survival benefit of approximately 3–6 months (4).

This Research Topic presents a remarkable collection of articles of 20 Original Research, 1 Case Report, 2 Reviews, and 1 Mini Review. The main purpose of this editorial is to introduce new prognostic factors for the GBM patients and to clarify newly discovered pathophysiological mechanisms of GBM. With the advances of molecular biology, molecular markers have gradually become an excellent diagnostic tool. In 2016, the WHO Classification of Tumors of the Central Nervous System (WHO CNS4) was the first to use molecular markers in glioma classification, and in 2021 WHO CNS5 placed more emphasis on their importance (5, 6). Several major genomic alterations had been identified. Epidermal Growth Factor Receptor (EGFR) amplification/mutations, Phosphatase and tensin homolog (PTEN) deletion/mutations and CDKN2Ap16INK4a were most frequently observed in primary GBM (5, 7). IDH1 (Isocitrate dehydrogenase 1) mutation was identified as the most reliable diagnostic molecular marker of secondary GBM, while the mutation of IDH1 was correlated with an improved overall survival. Other than that, there are countless potential molecular markers being discovered.

Yan et al. found that the Fc Fragment of IgG Binding Protein (FCGBP) was highly expressed in glioma and was a poor prognostic biomarker. Besides, GSEA analysis revealed that FCGBP and co-expressed genes were mainly involved in the immune response and had a synergistic effect with immune infiltrating molecules and immune checkpoint members. Lin et al. performed GO analysis and KEGG analysis on the differential genes of normal and GBM tissues in multiple databases, and subnets of differential gene interactions were mapped to select the most significant molecules. Among them, TRAF3 interacting protein 3 (TRAF3IP3) significantly affected the OS of GBM patients. Biochemical assays were applied to reveal that TRAF3IP3 promotes glioma growth by affecting the ERK pathway. Radu et al. introduced specific isoforms, distribution and functions of Glial fibrillary acidic protein (GFAP) and discussed the role of GFAPd as a potential biomarker, and a possible therapeutic target in glioblastoma. Recently, with a better understanding of the biological behavior of GBM, the long-held dogma that GBM does not metastasize outside the brain has been overturned. Rong et al. reported an early-onset GBM patient with primitive neuronal component (GBM-PNC) who had developed systemic bone metastasis.

The major functions of the human immune system are to modulate organ homeostasis, produce protein against infectious pathogens, and remove damaged cells. Multiple research has shown that adaptive and innate immunity play indispensable roles in the onset of cancer, progression and treatment (8, 9). Thus, immunotherapy has become a revolutionary anticancer therapy over the past few decades. It had shown considerable benefits, such as enhancing survival in numerous cancers, such as lung cancer, melanoma, and breast cancer (10, 11). Immune cells within the tumor microenvironment play an important role in tumorigenesis. In addition to being used for the diagnosis and prognosis prediction of GBM, multiple newly discovered molecular markers are associated with the infiltration of immune cells.

Yuan et al. reported that *via* analyzing expression data and corresponding clinical data from public databases, including TCGA, GEO, and CGGA, KDEL endoplasmic reticulum protein retention receptor 1 (KDELR1) was found upregulated in glioma samples and significantly correlated with poor prognosis and multiple clinical characters (age, recurrence, necrosis, microvascular proliferation, IDH mutation, and 1p/19q codeletion status). Moreover, KDELR1 expression level was positively associated with immune infiltration and microenvironment parameters. Likewise, Jiang et al. found that the expression of Non-SMC condensin I complex subunit G (NCAPG) was higher in glioma and correlated with unfavorable clinical characteristics. They found that NCAPG was correlated with tumor infiltration of B cells, CD4+ T cells, CD8+ T cells, neutrophils, macrophages and dendritic cells. Zheng et al. also demonstrated that the expression of NCAPG could be an independent prognostic factor of GBM patients and by using the CIBERSORT algorithm in 22 subpopulations of immune cells in glioma tissue, they found that NCAPG significantly negatively correlated with natural killer cells. In addition, NCAPG was positively correlated with MHC-I molecules and ADAM17. Liu et al. described the biological characteristics of glioblastoma-associated macrophages and microglia, highlighting the emerging molecular targets and related signal pathways involved in the interaction between TAMs and glioblastoma cells, as well as the potential TAM-associated therapeutic targets for glioblastoma.

As the overall survival (OS) for GBM patients who receive the same treatment may differ significantly at the individual level, summarizing the different characteristics and identifying effective prognostic factors could stratify patients for personalized follow-up regimen development and further individualized improvement of prognosis. Chen et al. used bioinformatics approaches to build a prognostic risk model based on platelet-to-lymphocyte ratio (PLR) level, WHO grade, IDH mutation, and Ki-67 index. Scores of the model stratified GBM patients and showed prognostic value of serum inflammatory biomarkers including PLR. Zhu et al. conducted and validated a LGALS based novel nomogram of OS, progression-free survival (PFS), and disease-specific survival (DSS) with favorable predictive performance and might serve as a reliable prognosis model for survival. Galectin (LGALS) could promote the stemness maintenance of glioma stem cells (GSCs) and positively correlate with M2-tumor-associated macrophages (TAMs) infiltration, demonstrating the potential role of LGALS genes in glioma immunosuppression and immune escape. Huang et al. identified six immune-related genes (IRG) and conducted a predictive nomogram integrating the independent predictive factors to determine the OS of individuals with GBM, which had better predictive ability than any independent factor alone. Finally, they found that the expression levels of tenascin C (TNC) and somatostatin receptor 2 (SSTR2), two out of six IRGs, were confirmed to be significantly associated with patient prognosis by protein mass spectrometry and western blotting. Similarly, Wang et al. revealed that age, Karnofsky performance scores (KPSs), tumor location, glioma grade, glioma type, extent of resection (EOR), adjuvant chemotherapy (ad-CT), concurrent chemotherapy (co-CT), and IDH status were independent factors associated with short-term glioma recurrence They conducted a novel nomogram to predict the risk of short-term recurrence after surgery in glioma patients. In addition, they provided a free online prediction risk tool for this nomogram. Ma et al. used Weighted Gene Co-Expression Network Analysis (WGCNA), univariate regression, and lasso regression to obtain three immune-related signatures (IL1R1、TNFSF12 and VDR) and construct an immune-related prognostic model (IRPM) of 1,439 genes from ImmPort, InnateDB databases and TCGA. Furthermore, they found that IRPM could help clinicians identify patients who are sensitive to PD-1 immune checkpoint blockade. Bastos et al. evaluated the value of MVD-CD105 and Ki-67 as prognostic and therapy response biomarkers of 102 consecutive GBM patients treated with bevacizumab (the most used anti-angiogenic drug) upon recurrence between 2010 and 2017. CD105 plays a key role in angiogenesis and preferentially marks novel angiogenic vessels. It was a sensitive and specific biomarker of angiogenesis within the tumor. However, high expression level of MVDCD105 represented poor prognosis of recurrence GBM patients while portending no prognostic significance in the primary tumors. Ki-67 expression was not associated with differences in survival outcomes. Amino acid metabolic reprogramming is critical for maintaining the survival of cancer cells and modulating the surrounding microenvironment, which enhances the malignancy and immunosuppression of tumors. Xu et al. hypothesized that amino acid metabolism-related genes were potential GBM prognosis predictors and constructed a novel amino acid metabolism-related and immune-associated risk signature for predicting prognosis in glioma. Moreover, they identified two amino acid metabolism-related genes, proteasome 26S subunit, ATPase 5 (PSMC5) and proteasome 26S subunit, ATPase 3 (PSMD3), as novel biomarkers in GBM.

For better understanding and use of molecular markers, Ran et al developed an integrated and web-based database GlioMarker, the first comprehensive database for knowledge exploration of glioma diagnostic biomarkers, which provides accurate information on 406 glioma diagnostic biomarkers from 1559 publications. GlioMarker could provide rapid and comprehensive knowledge of glioma diagnostic biomarkers to facilitate high quality research and applications of clinicians and researchers.

Not limited to the exploration of biomarkers, several original articles also studied the pathophysiological mechanisms of glioma. Lin et al. reported that the RNA-binding motif protein 8A (RBM8A) is expressed highly in GBM and was able to promote GBM cells proliferation and invasion. In addition, they showed that RBM8A acts by facilitating the interaction between RBM8A-CBF1 (C promoter-binding factor 1), triggering NOTCH/STAT3 activation to promote GBM progression. Finally, they performed animal experiments *in vivo* and calculate the GSVA score to confirm that RBM8A expression provided good diagnostic accuracy. Qiu et al. demonstrated that the expression level of PRL1 (regenerating liver phosphatase 1) greatly increased in GBM and positively correlated with tumor grade. Moreover, they established that PRL1 enhanced GBM tumorigenicity and invasion ability *in vitro* and *in vivo* by epithelial-mesenchymal transition (EMT). Further, PRL1 stabilized Snail2 through the deubiquitination effect of USP36. Besides, PRL1 was a significant predictor of poor outcome proved by 26 glioblastoma patients’ specimens. Yang et al. reported that the expression level of circPIK3C2A was also higher in GBM and relative to GBM proliferation. They reported that circPIK3C2A acts as ceRNA for MiR-877-5p and MiR-877-5p suppressed GBM progression *via* targeting FOXM1. Xenograft tumor models established the function of the circPIK3C2A/miR-877-5p/FOXM1 axis. Wu et al. reported that lncRNA-GAS5 (growth arrest-specific transcript 5) expression was remarkably decreased in high-grade glioma and overexpression of lncRNA-GAS5 impaired the stemness and proliferation of glioma stem cells (GSCs). Moreover, they discovered that GAS5 inhibited the viability of glioma cells through miR-let-7e and miR-125a *via* stabilizing sperm acrosome associated 6 (SPACA6) from degradation. They also found that miR-let-7e and miR-125a directly block the IL-6/STAT3 pathway.

Biomarkers can be used not only to predict prognosis, but also to explore patient tolerance to drugs. Anti-angiotherapy (Bevacizumab) is currently regarded as a promising option for glioma patients who are resistant to temozolomide (TMZ) treatment. Shi et al. revealed that integrin subunit alpha 5 (ITGA5), regulated by methylation on two distinct sites, may predict dual-drug resistance to Temozolomide and Bevacizumab in glioma and lead to changes of cell morphology and polarity. Furthermore, it may increase the degree of malignancy and drug resistance of glioma by affecting vascular mimicry (VM) formation. Wang et al identified MxA (encoded by an interferon-stimulating gene MX1) as a potential biomarker for early recognition of responsive patients to heat shock protein peptide complex 96 (HSPPC-96), which has been proven to be a safe and preliminary effective therapeutic vaccine in treating newly diagnosed GBM. The authors speculated that a preexisting TCR clone was linked to the expression of MxA and prognosis of GBM patient.

In addition, the microbiome is highly associated with a wide array of central nervous system disease, and may affect the development, progression and therapy of GBM. The gut microbiome and intratumoral microbiome were discussed by Liang et al. in their review.

At the end of the Research Topic, one rare case report was presented. Maimaiti et al reported a rare adult case of symptomatic H3K27M-mutant multicentric GBM that presented in the brain, fourth ventricle, and cervical and lumbar spinal cord regions accompanied by acute pulmonary artery embolism. The authors emphasize that clinicians are supposed to perform an early MRI of the spinal cord for patients with a prior diagnosis of GBM with or without any new onset of signs and symptoms in the spinal cord to confirm the diagnosis at an early stage. The incidence of spinal metastasis of cerebral GBM may be increasing.

In summary, we are confident that while novel molecular markers are constantly harvested, biomarker targeted therapy in GBM will be a promising treatment option, but there is yet a long path forward to clinical application.

## Author contributions

All authors listed have made a substantial, direct, and intellectual contribution to the work and approved it for publication.
